# Comparison of Anterior Chamber Depth Measurements from the Galilei Dual Scheimpflug Analyzer with IOLMaster

**DOI:** 10.1155/2012/430249

**Published:** 2012-01-19

**Authors:** Roma P. Patel, Rahul T. Pandit

**Affiliations:** ^1^Methodist Eye Associates, The Methodist Hospital, Houston, TX 77030, USA; ^2^Department of Ophthalmology, Weill Cornell Medical College, New York, NY 10065, USA

## Abstract

*Purpose*. To compare anterior chamber depth (ACD), representing the distance between the anterior corneal surface and anterior lens surface measurements between the Galilei Dual Scheimpflug Analyzer and the IOLMaster. *Methods*. A retrospective review of 65 individual patient eyes with normal anterior segments, and no prior ocular surgery was performed. Patients underwent ACD measurements with both devices during the same session by a trained examiner. Interdevice agreement was evaluated using paired two-tailed *t*-tests, Pearson correlation coefficient, and Bland-Altman analysis. *Results*. The mean ± standard deviation (SD) ACD for the Galilei and IOLMaster was 3.37 ± 0.36 mm (range from 2.62 to 4.13) and 3.25 ± 0.38 mm (range from 2.34 to 3.92), respectively (Pearson correlation coefficient = 0.96). ACD mean difference was 0.12 mm (*P* < 0.0001); 95% limits of agreement was from −0.09 to 0.34. The Galilei measured slightly longer ACD values than the IOLMaster. There was no relationship between axial length and interdevice difference. *Conclusion*. ACD measurements correlate well between the Galilei and IOLMaster, with Galilei values on average 0.12 mm longer than the IOLMaster.

## 1. Introduction

An accurate evaluation of the anterior chamber depth (ACD—the measurement from the anterior surface of the cornea to the anterior surface of the crystalline lens) is critical when planning procedures utilizing a phakic or pseudophakic intraocular lens (IOL). Postoperative refraction is determined by several factors, including IOL power, axial length, Postoperative effective lens position (ELP), and corneal curvature [1–3]. Postoperative ELP is predicted and its calculation depends on preoperative ACD in some formulas, such as those developed by Olsen [3]. ACD can be measured by contact as well as noncontact methods. The limitations of conventional A-scan ultrasonography are that it requires operator-dependent axial placement of the probe and involves corneal contact resulting in corneal indentation, possibly leading to inaccurate results [4–6]. Whereas immersion A-scan avoids potential error due to corneal contact, it can be more time consuming. Noncontact methods of ACD measurement have become preferred due to their speed, relative ease of use, avoidance of topical anesthesia, and lack of corneal indentation.

The IOLMaster (Carl Zeiss, Jena, Germany) was the first FDA-approved device to utilize optical biometry based on partial coherence interferometry and has supplanted A-scan ultrasonography in measurement of axial length in most USA physicians' [7]. For ACD measurements, the IOLMaster uses a 0.7 mm-wide slit beam of light which is directed at a 30-degree angle into the anterior chamber. The instrument measures the distance between light reflections on the anterior corneal surface and the anterior crystalline lens surface [8]. Five serial measurements along the visual axis are averaged to determine the final ACD measurement.

The Galilei Dual Scheimpflug Analyzer (Ziemer, Port, Switzerland) is a noninvasive diagnostic system based on a rotating dual Scheimpflug camera integrated with a Placido topographer. It captures slit images from opposite sides of the illuminated slit and averages the elevation data obtained from corresponding opposite slit images [9]. This dual camera technique improves imaging of the cornea and better compensates for eye movement, optimizing pachymetry measurements and ACD measurement from the posterior corneal surface to the anterior crystalline lens.

This study evaluated the comparability of ACD measurements obtained from the Galilei and IOLMaster.

## 2. Materials and Methods

Institutional review board approval was obtained for this study from The Methodist Hospital Research Institute, Houston, TX, USA. A retrospective review was performed of patients at The Methodist Hospital, Houston, TX, USA from April through October 2010 who underwent ACD measurements on both the Galilei Dual Scheimpflug Analyzer (version 5.2.1) and the IOLMaster (version 5.5) on a single day. All devices were calibrated according to manufacturer recommendations prior to undertaking the measurements. All scans were performed on patients with cataracts who were scheduled for cataract surgery. Eyes were excluded if there was any prior history of ocular surgery or laser, corneal disease, glaucoma, uveitis, or retinal vascular disease. One-hundred five eyes of 65 healthy subjects met inclusion criteria. In patients for whom bilateral scans were performed, one eye from each patient was selected, randomizing between right and left eyes. A total of 65 eyes were included in this chart review.

All patients underwent examination with the Galilei first followed by the IOLMaster. All measurements for a given subject were taken during the same session by a single trained examiner. Examiners differed from session to session. Operation was performed according to manufacturers' guidelines. ACD measurements from the Galilei were compared to those obtained from the IOLMaster. The Galilei measures “internal” aqueous depth (AQD) from the posterior surface of the cornea, whereas IOLMaster measures ACD from the anterior corneal surface. Therefore, the Galilei central zone (1–4 mm) average corneal pachymetry value was added to the Galilei AQD measurement. Additionally, the axial length (AL) of the subjects' eyes was measured with the IOLMaster.

### 2.1. Statistical Analysis

Sample size calculation was performed using SigmaStat (Systat Software, Chicago, Ill, USA). With an estimated standard deviation of 0.2 mm, to detect a difference in mean ACD of 0.1 mm at a significance level of 5% and a power of 80%, a sample size of 64 eyes was required.

Statistical analysis was performed using Microsoft Office Excel 2007 Analysis ToolPak (Microsoft, Redmond, Wash, USA). Pearson correlation and paired two-tailed *t*-tests were calculated to compare ACD measurements between the two instruments. A probability of less than 5% (*P* < 0.05) was considered statistically significant. Agreement between the devices was evaluated using Bland-Altman analysis [10]. The 95% limits of agreement (LoA) was calculated using the mean difference ±1.96 standard deviation (SD). A scatter plot was created to evaluate the relationship between axial length and difference in ACD measurements between instruments.

## 3. Results

**Figure 1:**
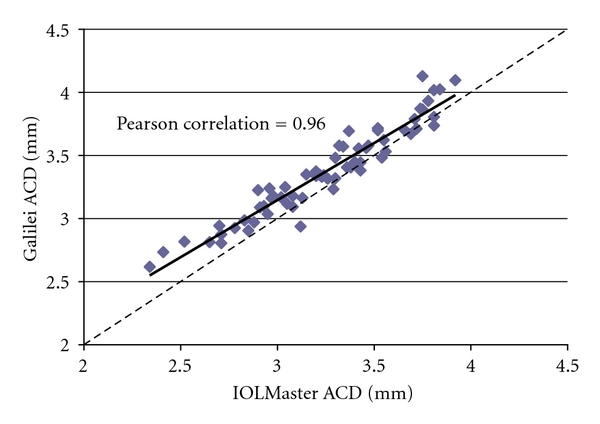
Comparison of ACD from IOLMaster versus Galilei. Anterior chamber depth (ACD) measurements in millimeters from the IOLMaster plotted against the Galilei reveal good correlation between the devices. The Galilei values are consistently higher than the IOLMaster values.

A total of 65 eyes of 65 patients were included; 36 were of males (55.4%), and 33 were left eyes (50.8%). Mean ± standard deviation (SD) patient age was  65 ± 14  (range from 20 to 91). The mean ± SD axial length was 24.5 mm ±1.62. The mean ± SD measurement for ACD on the Galilei and IOLMaster was 3.37 ± 0.36 mm (range from 2.62 to 4.13) and 3.25 ± 0.38 mm (range from 2.34 to 3.92), respectively (Table 1). ACD measurements between the two devices were well correlated (Pearson correlation coefficient = 0.96, Figure 1).

**Figure 2:**
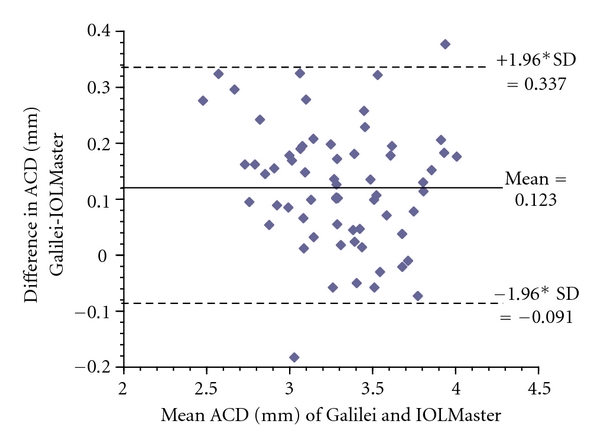
Bland-Altman plot of ACD: Galilei versus IOLMaster. Bland-Altman plot reveals that, on average, the Galilei measured longer anterior chamber depth (ACD) values than the IOLMaster by 0.12 mm (solid line), with a 95% limits of agreement (LoA) from −0.09 to 0.34 mm (dotted lines). Nearly all data lie within the 95% LoA and are evenly distributed, indicating no relationship between average ACD and interdevice difference.

**Figure 3:**
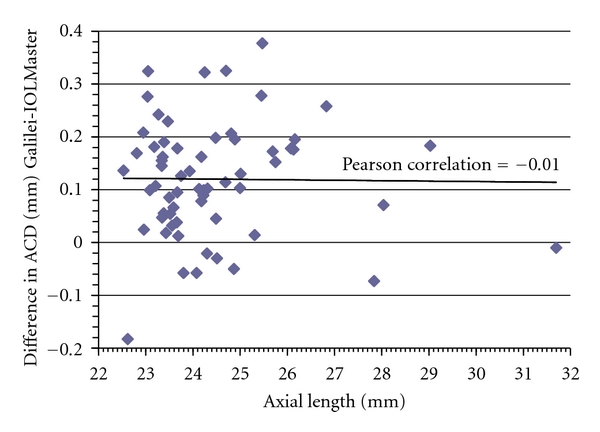
Axial length versus difference in ACD. Scatter plot depicting interdevice difference in anterior chamber depth (ACD) at various axial lengths (ALs) reveals evenly distributed data points, and thus no relationship between the two variables.

 Mean difference ± SD in ACD between Galilei and IOLMaster was 0.12 ± 0.11 mm; the 95% limits of agreement (LoA = ±1.96 SD) was moderate, ranging from −0.09 to 0.34 mm (Figure 2). Paired *t*-test revealed there was a statistically significant difference between the devices (*P* < 0.0001, Table 1). The Galilei measured slightly longer ACD measurements than the IOLMaster. There was no observed relationship between mean ACD and interdevice difference in ACD (Figure 2). A scatter plot depicting difference in ACD measurements between devices as a function of axial length (Figure 3) revealed no relationship between the two variables (Pearson correlation coefficient = −0.01).

## 4. Discussion

Obtaining accurate ACD measurements is critical for success with cataract surgery and intraocular lens implantation when using the Haigis, Holladay 2 or Olsen formulas, or ray tracing for power calculation. In this study, we compared ACD measurements obtained using two devices: the Galilei Dual Scheimpflug Analyzer and the IOLMaster. The mean ACD measurements between the two devices were closely correlated. Though there was a statistically significant difference in mean ACD between devices, this difference was clinically very small. The Galilei measurements were on average slightly longer than the IOLMaster.

The Galilei is unique in that it utilizes two Scheimpflug cameras to better account for eye movement during examination and theoretically obtains results of higher accuracy. This differentiates the Galilei from the Pentacam (Oculus, Wetzlar, Germany), which incorporates a single Scheimpflug camera. In a recent study, the Galilei ACD values have been found to be similar to the Pentacam though both were significantly less than the Orbscan II (Bausch and Lomb, Rochester, NY, USA), a scanning slit beam device, by approximately 0.3 mm [9].

The Pentacam has been available for a longer period of time and therefore has been more extensively studied. Results comparing it to other traditional devices that measure ACD have yielded mixed results. In one study, ACD measurements on the Pentacam differed significantly from the IOLMaster and ultrasound, with longer values on the Pentacam, though by approximately only 0.1 mm [8]. Reuland and colleagues found an even smaller difference between Pentacam and IOLMaster [11]. Savini and colleagues theorize that the small difference between the Scheimpflug and ultrasound measurements occurs due to a misidentification of the anterior edge of the anterior lens capsule with the Pentacam [12]. On the other hand, Nemeth and colleagues found no significant difference between Pentacam and traditional ultrasound in phakic eyes; they did find that the Pentacam measured significantly smaller ACD measurements than ultrasound in pseudophakic eyes [13]. It should be noted, however, that the Pentacam software has undergone multiple upgrades, and differences will be found depending on the software versions. Past studies have also shown variable results when comparing a different Scheimpflug device (EAS-1000, Nidek, Gamagori, Japan) with ultrasound [5, 14].

Our data is consistent with the above studies comparing single-camera Scheimpflug imaging with IOLMaster though we are unaware of any prior report evaluating dual-Scheimpflug imaging of the Galilei with the IOLMaster. In our study, nearly all measurements between the two devices were within 95% LoA. The LoA range was 0.43 mm, consistent with prior studies of ACD [8, 9, 15]. The mean difference in ACD of 0.12 mm between our devices, though statically significant, was very small. Using the Haigis formula, with an axial length of 23.5 mm and an average keratometry of 44 D (diopters), a difference in ACD of 0.12 mm would result in a change in the target refractive error by only 0.06 D for a common posterior chamber IOL (whereas the same error in axial length would cause a 0.31 D target refractive error).

This study is limited by its retrospective design and the use of multiple examiners between sessions. Though good repeatability has been demonstrated on the Galilei using a single examiner [16], interobserver variability has not been addressed. Furthermore, agreement with another conventional device such as ultrasound was not performed, and therefore comments on accuracy of data from the two devices can only be interpreted relative to each other. Additionally, accuracy of the ACD measurement from the Galilei is dependent on the accuracy of its measurement of central corneal thickness. Without a separate evaluation of the validity of the pachymetry values from the Galilei, it is not possible to determine if the interdevice difference was due to variability in pachymetry or internal AQD measurement. The peer-reviewed literature is varied in its outcomes when comparing Galilei central pachymetry values with other optical devices and ultrasound; reported mean differences have ranged from 0 to 27 microns [17–20]. Such differences in pachymetry values would at best, however, only account for a very small portion of the interdevice difference in ACD observed in our study.

In summary, our data demonstrates good correlation in ACD between the Galilei and IOLMaster. The Galilei mean ACD values were on average 0.12 mm longer than the IOLMaster, with a narrow 95% LoA interval of 0.43 mm. Additional studies evaluating these two devices against other instruments are recommended.

## Figures and Tables

**Table 1:** Summary of ACD measurements and mean interdevice difference between Galilei and IOLMaster.

Device	Mean (range) ACD	SD
Galilei	3.37 (2.62–4.13)	0.36
IOLMaster	3.25 (2.34–3.92)	0.38
Galilei-IOLMaster	0.12*	0.11**

*P < 0.0001

**95% LoA −0.09 to 0.34

ACD: anterior chamber depth; SD: standard deviation; LoA: limits of agreement. All measurements in millimeters.

Mean ACD, SD, and range of values are shown above for the Galilei and IOLMaster. Mean interdevice difference in ACD is statistically significant.
